# Ultrasound Strain Elastography Reliability in the Assessment of the Plantar Fascia and Its Relationship with the Plantar Thickness in Healthy Adults: An Intra and Interobserver Reliability Study in Novice Evaluators

**DOI:** 10.3390/biomedicines11072040

**Published:** 2023-07-20

**Authors:** Daniel Aguilar-Nuñez, Pablo Cervera-Garvi, Maria Aguilar-Garcia, Maria Cuevas-Cervera, Ana Gonzalez-Muñoz, Santiago Navarro-Ledesma

**Affiliations:** 1 Department of Nursing and Podiatry, Faculty of Health Sciences, University of Malaga, Arquitecto Francisco Penalosa 3, Ampliación de Campus de Teatinos, 29071 Malaga, Spain; daguilarn.tic@gmail.com; 2 Clinica Ana Gonzalez, Avenida Hernan Nuñez de Toledo 6, 29018 Malaga, Spain; maaricuevass@correo.ugr.es (M.C.-C.); agonzalezm@correo.ugr.es (A.G.-M.); 3 Department of Physiotherapy, Faculty of Health Sciences, Campus of Melilla, University of Granada, Querol Street, 5, 52004 Melilla, Spain; maguilar.fisioterapia@gmail.com (M.A.-G.); snl@ugr.es (S.N.-L.)

**Keywords:** plantar fascia, ultrasound, sonoelastography, intraobserver, interobserver, reliability

## Abstract

Purpose. This study was aimed at verifying both the intraobserver and interobserver reliability of measuring plantar fascia stiffness for a given image in healthy active adults. Methodology. This study is reported following the Guidelines of Reporting Reliability and Agreement Studies. A total of 20 plantar fascia from healthy volunteers were analyzed. The thickness of the plantar fascia was measured vertically from the anterior edge of the inferior calcaneal border to the inferior border of the plantar fascia and the ultrasound elastography measurement was taken at the calcaneal insertion of the plantar fascia with the region of interest one centimeter away from the insertion. Results. The ultrasound strain elastography measurements: the right intraobserver 1 showed an ICC value of 0.9 and the left intraobserver 1 showed an ICC value of 0.78, while the right intraobserver 2 showed an ICC value of 0.91 and the left intraobserver 2 showed an ICC value of 0.83. Interobserver measurements showed excellent reliability with a right ICC value of 0.8 and a left ICC value of 0.9 for the plantar fascia thickness measurements. Discussion. The results of this study showed a strong correlation between left and right plantar fascia thickness. The intraobserver reliability was excellent for both plantar fascia ultrasound strain elastography and thickness measurements, with interobserver measurements showing excellent reliability.

## 1. Introduction

The plantar fascia (PF), which is a deep, strong, fibrous, thick aponeurotic connective tissue, gives the foot’s medial longitudinal arch stability. It frequently takes the form of a stocking-like structure made up of sheets, which surrounds tendons and muscles under the superficial fascia [1]. It has its origin at the medial process of the calcaneal tuberosity and reaches out towards the toes in three distinct structural bands, namely: the lateral, medial, and central. The largest is the central area, which is the most susceptible to deformities and is the most affected by disease. The foot is stabilized by the PF during running and walking and thus it has an important role to play [2,3]. It decreases or increases the stiffness of the foot to mitigate external forces or to convey the extrinsic foot muscle’s internal force to the foot, respectively [4,5]. The common clinical presentation of plantar fasciopathy includes pain and discomfort, normally in the inferior heel region; however, it can also be associated with the radiation of pain along the entire foot as well. Plantar fasciopathy is a clinical manifestation that is usually accentuated with the first steps in the morning, and with sudden acute painful episodes in daily life [6,7,8]. The evaluation of PF disorders is important because ultrasound strain elastography is a new ultrasound-based imaging technique that provides information on tissue elasticity and stiffness, and it can be a useful objective method for the evaluation of plantar fascia pathology [9,10]. Diagnosis of plantar fasciopathy is generally based on clinical presentation and is often targeted with a multimodal management approach. In recent times, PF disorders have been evaluated by ultrasound strain elastography [11].

The musculoskeletal system can be assessed visually using radiological imaging techniques, which include computed tomography (CT), magnetic resonance imaging (MRI), and ultrasound (US) [12,13]. Ultrasound is a clinical and research device that is commonly used in injury diagnosis and prevention. A recent development is ultrasound strain elastography (SEL), which is a noninvasive ultrasound technique that permits the mechanical properties of tissues to be assessed in vivo and allows a noninvasive estimation of tissue stiffness [11]. The underlying principle of this technique is that a displacement or strain is produced in the tissue by compression. Two SEL methods are commonly used in musculoskeletal research and clinical practice: strain elastography, in which a mechanical force compresses the tissues axially, and shear wave elastography (SWE), in which compressive acoustic waves dynamically provide local stress in the soft tissues [14]. The principle underlying ultrasound strain elastography is that tissue compression produces a strain (displacement) within the tissue, which provides a color-coded image superimposed over the B-mode image. This provides a color-coded image, with the colors indicating the tissues’ relative elasticity in the region of interest (ROI), which is then superimposed on top of the B-mode image (a two-dimensional image in which the organs and tissues of interest are depicted as points of variable brightness) [9]. Ultrasound strain elastography is reported to be a reliable, valid, and useful tool to discern the thickness of the PF [15]. Sonoelastography has emerged as a useful imaging tool that can provide qualitative/quantitative assessment of tissue elasticity and promote the early diagnosis of musculoskeletal disorders, such as muscles, ligaments, joints injuries, and specifically tendinopathies, and is being widely in both musculoskeletal research and clinical settings. Key aspects that highlight the usefulness of elastography in musculoskeletal disorders are tissue characterization, differentiating between normal and abnormal tissues by quantifying their mechanical properties; injury assessment and monitoring; treatment guidance; and surgical planning, research, and rehabilitation [16,17]. However, its level of reliability is still too low due to the fact that measurements of PF stiffness are not standardized, resulting in reliability studies being difficult to generalize [18]. Furthermore, the examiners’ dependency on ultrasound means that their level of experience should also be considered. In this regard, the fact that novice examiners can be trained by experts and present reliable results must be studied.

The ultrasound strain elastography assessment of PF thickness has been reported to have a very good intraobserver and interobserver reliability [19]. The intraobserver and interobserver reliability using the Intraclass Correlation Coefficient (ICC) was 0.62 when one measurement was used and rose to 0.82 when an average of three measurements was used [19]. Intraobserver reliability, when one measurement was used, was 0.67 ICC and rose to 0.77 ICC when an average of three measurements was used. In addition, Salehi et al. reported on intraobserver reliability for plantar fascia thickness and echogenicity with ICCs of ≥0.89 and ≥0.89 being found, respectively, for the healthy controls, while the plantar fasciitis group had values of ≥0.87 and ≥0.90, respectively [20]. The intraobserver and interobserver reliability of SEL in PF has been reported in the literature. Rios et al. showed an interobserver reliability of 0.524 with the intraobserver reliability being 0.672 [9]. Additionally, Wu et al. reported on the reliability of SEL, showing an ICC of 0.765 for interobserver reliability and an ICC of 0.818 for the intraobserver reliability [21].

The sources of errors found when repeatedly taking measurements via ultrasound in the assessment of PF in healthy adults come from visually inspecting the image characteristics, such as application, imaging modality, human interaction, the homogeneity of images, spatial characteristics of images, continuity, texture, and image content [2]. Nowadays, there is still a lack of studies which determine whether the SEL assessment of PF can be carried out by novice evaluators. Therefore, this study was aimed at verifying both the intraobserver and interobserver reliability of measuring plantar fascia stiffness for a given image in healthy active adults, with the measurements being carried out by two newly trained evaluators under expert supervision, to show whether novice US examiners are still able to produce reliable results or not. Finally, another objective was to study the PF thickness and the level of association with the stiffness of the PF for a given image in healthy active adults.

## 2. Materials and Methods

### 2.1. Testing

This study is reported following the Guidelines of Reporting Reliability and Agreement Studies (GRRAS) [22] and approved by the Ethics Committee of the University of Málaga (CEUMA Registration numb er: 101-2022-H). This study was aimed at building validity, measurement variability, and interobserver reliability analysis. This type of study is centered on assessment based on the accumulation of evidence using a specific measuring instrument (e.g., quantitative elastography).

### 2.2. Training Phase

Prior to the intra–interobserver reliability study, two novice US observers were trained for a period of 3 weeks under the supervision of an expert with 11 years of experience in US and SEL. This training phase consisted of lectures, clinical demonstrations, and supervised clinical practices focused on the plantar facia SEL assessment. Subsequently, the SEL measurements were started when the two novice US observers had finished the training phase.

### 2.3. Participants

A total of 20 plantar fascia from healthy volunteers were analyzed. Volunteers were recruited from a private polyclinic in Malaga (Spain). To summarize, the participants’ demographic data were age 36.6 (years), height 1.77 (m), body mass 78.05 (kg), BMI 25.13 (kg/m^2^), FP thickness 4.13 (mm), plantar fascia elasticity 2.23 (Young’s modulus: 1/4 stress/strain). The inclusion criteria were: (i) having no injuries in the lower limb in the past two years, (ii) being physically active (150 min of moderate-intensity or 75 min of vigorous-intensity aerobic exercise weekly), (iii) being aged between 18 and 55 years of age, and (iv) having no pain or discomfort at the moment of evaluation. The exclusion criteria were as follows: (i) diagnosis of a systemic inflammatory illness, (ii) diagnosis of connective tissue disorder, (iii) previous local trauma, (iv) existence of plantar fibroma, and (v) treatment with plantar fascia hyaluronic acid, corticosteroids, or injections of plasma rich in platelets within the 3 months prior to screening. The participants were fully informed of the characteristics of the study before providing signed consent.

### 2.4. Procedures

The US imaging was conducted by means of a GE Logiq-S7 and a 3.0–10.0 MHz linear-array transducer (GE Healthcare) with a frequency of 8 MHz and “coded harmonic imaging” [23] was used for the study interval. Nevertheless, the US examiner could change the depth, gain, or focus as necessary. All the plantar fascia thickness and SEL measurements were taken in millimeters. Participants were positioned in ventral decubitus on a flat table, with their feet hanging beyond the table edge [18]. Each subject was examined while lying prone with 90 degrees of knee flexion in the neutral ankle position and the central band of the plantar fascia of the foot was traced by hand from the arch to the heel in order to discern the borders. The fascia was first assessed by B-mode ultrasound for thickness and echotexture. In a longitudinal view, the thickness of the plantar fascia was measured vertically from the anterior edge of the inferior calcaneal border to the inferior border of the plantar fascia [24]. The maximum thickness (in mm, craniocaudal dimension) of each plantar fascia was measured in the longitudinal plane (i.e., perpendicular to the direction of the fibers) at the insertion point of the plantar fascia at the calcaneus.

**Figure 1:**
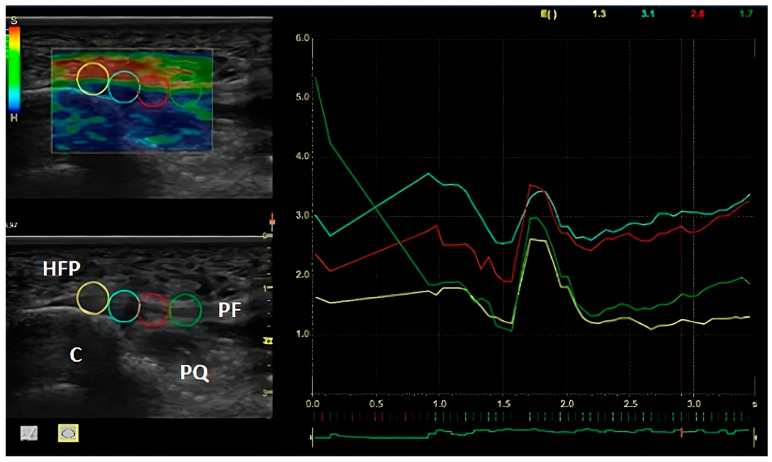
SEL measurements. Region of interest (ROI) and SEL measurements. Note: Left to right: Point 1: PF insertion to the calcaneus; Point 2: body of the FP in mid-proximal portion; Point 3: body of the FP in middle portion; Point 4: body of the PF in mid-distal portion. Note: The anatomic structures on the ultrasound imaging: HFP (heel fat plantar), PF (plantar fascia), C (calcaneus), PQ (plantar quadratus).

Plantar fascia was located using the insertion to the calcaneus as the upper border and the body of the PF in mid-distal portion as the lower border. The selected image featured 5 green bars, with this indicating the highest level of quality recommended by the inbuilt software in the computer. Four circular 5 mm regions of interest (ROI), which ranged from the insertion to the calcaneus as the upper border to the body of the PF in mid-distal portion, were used to calculate the SEL value along the plantar fascia (see Figure 1). The values are shown through a plot ranging from 0 to 6, from the softest to the hardest. The stiffness color scheme was red (hard), green (medium), and blue (soft). The regions of interest to be measured, are shown in different colors. The quantitative analyses to measure the plantar fascia SEL is shown through a plot, showing the corresponding values, ranging from 0 to 6, using the same colors as those shown in the different ROI. Furthermore, a scale bar is also represented, where the softest tissue is shown in red and is represented by the letter “S” on the scale bar’s top, and the hardest tissue is shown on blue, and represented by the letter “H” on the scale bar’s bottom.

**Figure 2:**
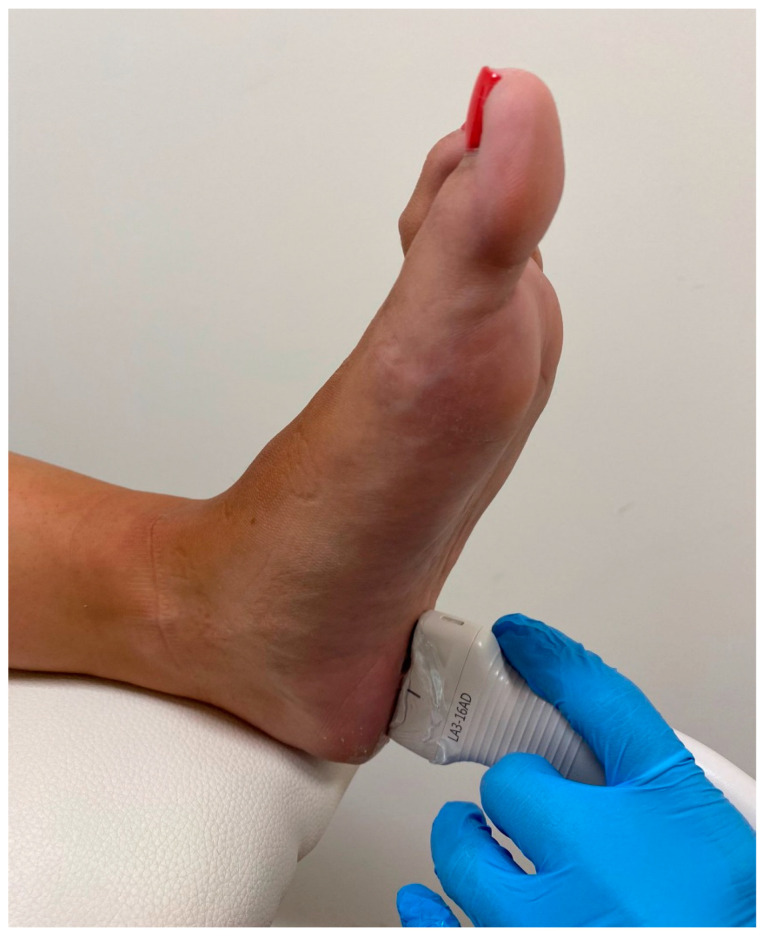
Transducer positioning during the plantar fascia SEL assessment.

Imaging of plantar fascia consisted of three independent real-time scans per subject. For each scan, one real-time scan was chosen, resulting in three images per subject (average measurement of three scans). A water-based clinical gel layer was used for the acoustic coupling between probe and tissue to minimize pressure over the skin (Figure 2).

### 2.5. Data Analysis

Statistical analysis was performed using the SPSS software version 26.0 (IBM Corp., IBM SPSS Statistics for Windows, Armonk, NY, USA). The Intraclass Correlation Coefficient (ICC) was used to analyze the measurement reliability with the two-way mixed model on single measurement and absolute agreement used for intraobserver and interobserver measurements. The ICC has values between 0 and 1 where higher values indicate greater reliability, hence a value from 0 to 0.40 is considered poor, from 0.40 to 0.59 is considered regular, from 0.60 to 0.75 is considered good, and from 0.75 to 1.0 is considered excellent. The mean of three trials of one rater is considered as the assessment basis. For both intra- and inter-rater reliability, the mean of three trials was reported. The intraobserver and interobserver variability was analyzed by making 3 consecutive measurements for each observer and repeating the same measurements 10 days after the first measurement. Confidence intervals (CI) of 95% were used. In addition, the Cronbach’s alpha was calculated to determine the internal consistency, which according to Bland and Alman et al. [25] should optimally be above 0.9 in clinical practice, which is within the range possible from 0 to 1. A paired Student’s *t*-test was performed for related samples to test the hypothesis. Pearson’s correlation test was used to study the relationship of the plantar fascia thickness and SEL measurements. Strong correlation was defined as values greater than 0.7; between 0.5 and 0.7 correlation was considered moderate; between 0.3 and 0.5 was considered low correlation; and a *p*-value < 0.05 was considered statistically significant.

## 3. Results

A total sample of 20 PF were analyzed. The average age of the population included was 30.6 years and was made up of the same number of women as men. The BMI of the participant set was 25.13 (kg/m^2^), as can be seen in Table 1. The plantar fascia thickness measurements for right and left intraobserver 1 reliability were excellent, showing ICC values of 0.98 and 0.97, respectively. As regards the SEL measurements, the right intraobserver 1 showed an ICC value of 0.9 and the left intraobserver 1 showed an ICC value of 0.78. In addition, the plantar fascia thickness measurements for right and left intraobserver 2 reliabilities were excellent, showing ICC values of 0.93 and 0.93, respectively. Regarding the SEL measurements, the right intraobserver 2 showed an ICC value of 0.91 and the left intraobserver 2 showed an ICC value of 0.83 (Table 2).

In Table 3, interobserver reliability is shown. Interobserver measurements showed excellent reliability, with a left ICC value of 0.9 and a right ICC value of 0.8 for the plantar fascia thickness measurements and left ICC values of 0.47 and right ICC values of 0.49 for the SEL measurements.

In Table 4, Pearson correlations between SEL and thickness values are shown. However, the level of association between the plantar fascia thickness and the plantar fascia stiffness for a given image in healthy subjects showed a low level of association in both observers (*r*-values < 0.3), with values between 0.0899 (*p* = 0.636) to 0.4433 (*p* = 0.0141) and 0.0457 (*p* = 0.810) to 0.2004 (*p* = 0.288) for observer 1 and observer 2, respectively.

## 4. Discussion

This study had the goal of analyzing the reliability, both intraobserver and interobserver, of plantar fascia stiffness and thickness measurements for a given image in healthy active adults, carried out by two novice observers. In addition, it aimed to examine the level of association between the plantar fascia’s thickness and stiffness. Both the intraobserver 1 and 2 reliabilities for plantar fascia thickness measurements were excellent. The plantar fascia thickness measurements’ interobserver reliability was excellent too. Finally, the level of association between the PF thickness and the plantar fascia stiffness for a given image in healthy subjects showed a low level of association in both observers (*p*-values > 0.0001).

Nowadays, although studies on plantar fascia and the reliability of SEL measurements in healthy subjects exist, there is more research to be done in order to get a consensus. In healthy volunteers, the plantar fascia SEL results were stated as 5.4 (0.6) m/s by Chino et al. [26], 148 m/s, 111.9 m/s, and 90.0 m/s by Wu et al. [21] using conventional strain elastography. It is possible that the differences in the results between studies were due to the SEL methods commonly used in musculoskeletal research and clinical practice, namely strain elastography and shear wave elastography (SWE). In this regard, our results showed similar values when assessing SEL in healthy plantar fascia; nevertheless, the level of association between PF elasticity and thickness was very low. In the study by Ríos et al. [9], which was conducted in healthy patients and in patients with symptomatic PF using conventional strain elastography (SEL), the appearance of the PF was evaluated for reproducibility in image acquisition. The intraobserver reliability demonstrated an ICC value of 0.91 (95% CI) on the left and 0.82 (95% CI) on the right, while interobserver measurements showed good reliability with an ICC of 0.81 and 95% CI. SEL interobserver reliability of the PF showed an ICC that was moderate because the PF is a deep anatomical feature. Hence, the way the SEL ultrasound attenuates the soft tissue that overlies this area, which includes heel fat and hyperkeratotic skin, will probably have a greater influence on the way the image is seen [9]. In addition, Sconfienza et al. [11] showed a change in the viscoelastic properties of the PF of the injured group upon comparison with those of the healthy group when using conventional strain elastography. In recent studies, the use of conventional SEL has already been demonstrated, with results of 146.9 kPa in the healthy volunteer group and 129.4 kPa in the group with injuries [9,21,27]. The loss of elasticity indicates disease progression and/or the effects of non-successful aging. In these studies [9,21], there was an injured group with plantar fasciopathy and SEL data was obtained by examiners with 3 to 10 years of experience [9,21]. Zhang’s elastography study showed 5.52 m/s (decrease) SEL values [28] and Shiotani’s SEL study showed 8.0–9.5 m/s (increase) SEL values [29]. The difference between these studies may be due to the fact that Zhang et al. [28] used only elderly subjects and SWE measurements and Shiotani et al. [29] recruited only physically inactive people and used conventional strain elastography; and additionally, as a neutral stationary placement was used to examine the ankle, both studies probably measured the elasticity of the tissue with the PF being tighter [30]. Previously, a qualitative method using histogram comparisons of the different color distributions was frequently employed for image analysis when SEL was used in PF examinations. The ultrasound system is provided with a color scale, in which blue corresponds to the most elastic tissue and red to the least elastic tissue, with green as the intermediate value [11]. These studies demonstrated that SEL of the plantar fascia, which was represented by a blue color on the ultrasound, were in general firm structures in the healthy subjects. In contrast, a uniform red color was seen for the PF of those younger subjects, while various sparse areas of yellow or green were observed amid the red color in older volunteers, thus indicating areas of moderate stiffness. The aforementioned studies were not able to quantitatively measure the elasticity of the fascia, however quantitative studies showed that human tendons and ligaments have a similar elasticity to the plantar fascia [31]. In addition, a recent meta-analysis [23] study showed that the PF were less stiff in the plantar fasciopathy group than in asymptomatic subjects, and other studies have shown PF from subjects suffering from plantar fasciitis to be softer than those in healthy controls [32,33,34]. In this regard, our results showed similar values when assessing SEL in healthy plantar fascia; nevertheless, the level of association between PF elasticity and thickness was very low. If this relationship were found, it would be of great interest to both clinicians and researchers since the loss of elasticity indicates disease progression and/or the effects of non-successful aging [35]. Although the presented results showed no relationship between PF elasticity and thickness, further studies should analyze this not only in healthy subjects, but also in those suffering from chronic PF pain. In this line, current studies have found a relationship between elastic properties of tissue and alterations in the autonomic nervous system [35,36]. This is only a hypothesis and more studies in this line are needed.

In the literature, the reliability of PF thickness has been previously reported in subjects between 22 and 76 years of age, both healthy and with plantar heel pain, showing values of high reproducibility (ICC 0.76–0.94) [37]. In the study by Skovdal et al., which used 20 healthy subjects between the ages of 20 and 31 and with no symptoms in the lower extremities, it was shown that the mean of three measurements instead of just one single measurement increases the reliability. Thickness changes that are >0.6 mm can be thought of as real thickness changes, according to the intratester reliability ‘limits of agreement’, and not thought of as a measurement error [19]. In our reliability study, which used 20 healthy subjects between the ages of 22 and 42 years of age, a non-pathological PF thickness of 4.13 (0.103) mm in the participants was reported.

Currently, there is a variation in the ultrasonically measured value of the plantar fascia’s normal thickness; Cardinal et al. [38] reported its mean to be 2.6 mm (1.6–3.8 mm) while Long et al. [39] reported its mean as 3.3 mm (2.4–4.3 mm). The values for men and women were shown to be 3.6 mm and 3.4 mm, respectively, in the study by Wall et al. [40]. Our results reported a PF thickness of 4.13 mm (0.103) visible on B-mode imaging using US and a stiffness of 1.636 (0.913) measured by SEL. Sonography has been previously used to research various plantar fascia echogenicity and thickness measurements as regards both absolute and relative reliability. In contrast, Wu et al. [21] demonstrated in their study using 20 symptomatic plantar fascia in 13 subjects that the interobserver reliability of ICC was 0.792. Upon examination using a traditional US technique, the plantar fascia thickness was found to be significantly thicker in the group with fasciitis when compared to that of the healthy older volunteers (3.7 mm vs. 2.7 mm, *p* < 0.001) [21]. In addition, several studies have reported a 2.26 mm PF thickness at a distance of 3 cm distal to its insertion point on the medial calcaneal tubercle when the mean of three measurements from three sonographs was used [41]. Echogenicity reliability was shown to be excellent for all three measurement positions (ICC > 0.90) [41] with the methodology and clinical training being similar to ours. Narindra et al. [42] studied 226 feet and showed the PF thickness to be symmetrical (average 3 mm ± 0.5) in addition to demonstrating it has a good correlation with BMI, age, height, daily walking, and weight (*p* < 0.05). Cheng et al. [37], who studied the reliability and reproducibility of measurements of plantar fascia echogenicity and thickness, showed a PF thickness of 0.32 cm. This study produced results that showed a strong correlation regarding the thickness of the plantar fascia on the right and left; this is harmonious with earlier studies [41,42].

The potential and usefulness of using elastography in plantar fasciitis diagnosis is widely known [11]. In this regard, some examples of its use are early plantar fasciitis detection [27], which is demonstrated by the afflicted side having plantar fascia softening [11], and decreased stiffness in microchambers, macro chambers, and heel pad [27].

### 4.1. Clinical Significance of the Results

All the aforementioned assessments from previous studies were carried out by expert evaluators. In contrast, all measurements from our study were carried out by novice evaluators after a period of training (3 weeks) from an expert with 10 years of experience in ultrasound imaging. Our results show that the PF can be assessed by novice evaluators after proper training. In addition to the use of US in the evaluation of patients, SEL appears to be a noteworthy tool in this regard. Additionally, the efficacy of rehabilitation and treatment protocols can be usefully monitored by ultrasound strain elastography, which yields quantitative data and, as regards functional and pain scores, demonstrates a reliable correlation [18]. In order to raise ultrasound measurement reliability and diminish mistakes, it is essential to observe the evidence that sonogram acquisition parameters, such as the positioning of the probe and its orientation, are crucial [18].

### 4.2. Strengths and Weaknesses of the Study

Some strengths of this study must be reported. This is the first study in which the examiners were novices, and after a period of training were able to obtain good reliability results, showing that assessments may be carried out by health professionals in their early careers. In addition, the measurements were not blinded.

However, several weaknesses have to be acknowledged. The sample size was small and the participants were healthy subjects; thus the presented results, as well as any extrapolation to other populations, should be interpreted cautiously.

Further research is suggested to improve knowledge in this field, which could include increasing the number of participants and/or including a pathological population to be able to compare intra—interobserver data in both novice and expert evaluators.

## 5. Conclusions

The intraobserver reliability was excellent for both PF elastography and thickness measurements, with interobserver measurements showing excellent reliability. There was a strong correlation between left and right PF thickness and SEL in healthy subjects and those assessments can be reliably carried out by novice evaluators.

## Figures and Tables

**Table 1:** Anthropometric characteristics of the sample.

	Mean (SD) N = 10 (20 Feet)	Mean Man/Woman
Man/Woman	5/5	
Age (years)	30.6 (5.85)	30.4/30.8
Height (m)	1.75 (9.05)	1.81/1.70
Body mass (kg)	78.05 (22.56)	86.4/69.7
BMI (kg/m^2^)	25.13 (5.49)	26.4/24.12
Daily sport (min)	44 (13.90)	42/46
Physical activity level (%)	Light 50% Moderate 40% Intense 10%	60%/40% 20%/60% 20%/0%
FP thickness (mm)	4.413 (0.103)	4.48/4.34
Plantar fascia elasticity, mean (SD) (Young’s modulus: 1/4 stress/strain)	2.239 (1.005)	2.372/2.106

Note: Mean (SD). BMI: body mass index; m: meters; min: minutes; cm: centimeters.

**Table 2:** Intraobserver reliability and Cronbach’s alpha values.

	**Observer 1 (Right)**	**Observer 1 (Left)**	**Observer 2 (Right)**	**Observer 2 (Left)**
Intraobserver reliability				
Plantar fascia thickness (95%CI)	0.93 (0.80–0.98)	0.97 (0.89–0.99)	0.93 (0.82–0.98)	0.98 (0.94–0.99)
Elastography (95%CI)	0.83 (0.63–0.95)	0.78 (0.54–0.93)	0.91 (0.80–0.97)	0.90 (0.77–0.97)
	**Observer 1 (Right)**	**Observer 1 (Left)**	**Observer 2 (Right)**	**Observer 2 (Left)**
Cronbach’s alpha				
Fascia thickness (cm)	0.973	0.993	0.980	0.994
Elastography (Young’s modulus: 1/4 stress/strain?)	0.902	0.855	0.913	0.900

Note: 95% CI: 95% confidence interval; SD: standard deviation. ICC: intraclass correlation; cm: centimeters.

**Table 3:** Interobserver reliability.

	Plantar Fascia Thickness		Plantar Fascia Elastography	
	ICC (95% CI)	Mean Difference (SD)	ICC (95% CI)	Mean Difference (SD)
Interobserver reliability right	0.802 (0.233–0.951)	0.826 (0.185)	0.494 (0.242–0.790)	2.038 (0.729)
Interobserver reliability left	0.932 (0.765–0.982)	0.835 (0.227)	0.476 (0.221–0.781)	2.383 (0.639)

Note: Interobserver reliability of plantar fascia thickness value and interobserver reliability of plantar fascia SEL value. 95% CI: 95% confidence interval; SD: standard deviation.

**Table 4:** Pearson correlations between SEL and thickness values in observer 1 and Pearson correlations between SEL and thickness values in observer 2.

**Intraobserver 1**				
	**Plantar Fascia SEL (Right)**	**Plantar Fascia SEL (** **Left)**	**Plantar Fascia Thickness (** **Right)**	**Plantar Fascia Thickness (** **Left)**
Plantar fascia SEL (right)		0.2537 (0.1762)	0.0899 (0.6365)	0.4433 (0.0141)
Plantar fascia SEL (left)	0.2537 (0.1762)		−0.0887 (0.6413)	0.1663 (0.3797)
Plantar fascia thickness (right)	0.0899 (0.6365)	−0.0887 (0.6413)		0.7711 (<0.0001)
Plantar fascia thickness (left)	0.4433 (0.0141)	0.1663 (0.3797)	0.7711 (<0.0001)	
**Intraobserver 2**				
	**Plantar Fascia SEL (Right)**	**Plantar Fascia SEL (Left)**	**Plantar Fascia Thickness (Right)**	**Plantar Fascia Thickness (Left)**
Plantar fascia SEL (right)		0.4573 (0.0111)	0.0457 (0.8104)	0.1245 (0.5121)
Plantar fascia SEL (left)	0.4573 (0.0111)		0.0708 (0.7099)	0.2004 (0.2883)
Plantar fascia thickness (right)	0.0457 (0.8104)	0.0708 (0.7099)		0.9222 (<0.0001)
Plantar fascia thickness (left)	0.1245 (0.5121)	0.2004 (0.2883)	0.9222 (<0.0001)	

Note: Level of association between the plantar fascia thickness and the plantar fascia stiffness for a given image in healthy subjects.

## Data Availability

The data are available to consult.
